# The methods for removal of direct oral anticoagulants and heparins to improve the monitoring of hemostasis: a narrative literature review

**DOI:** 10.1186/s12959-023-00501-7

**Published:** 2023-05-19

**Authors:** Aleksandra Frackiewicz, Bartlomiej Kalaska, Joanna Miklosz, Andrzej Mogielnicki

**Affiliations:** Department of Pharmacodynamics, Medical University of Bialystok, Bialystok, Poland

**Keywords:** Anticoagulants, Diagnostic tests, Direct oral anticoagulants, Heparin, Neutralization

## Abstract

The assessment of hemostasis is necessary to make suitable decisions on the management of patients with thrombotic disorders. In some clinical situations, for example, during thrombophilia screening, the presence of anticoagulants in sample makes diagnosis impossible. Various elimination methods may overcome anticoagulant interference. DOAC-Stop, DOAC-Remove and DOAC Filter are available methods to remove direct oral anticoagulants in diagnostic tests, although there are still reports on their incomplete efficacy in several assays. The new antidotes for direct oral anticoagulants – idarucizumab and andexanet alfa – could be potentially useful, but have their drawbacks. The necessity to remove heparins is also arising as heparin contamination from central venous catheter or therapy with heparin disturbs the appropriate hemostasis assessment. Heparinase and polybrene are already present in commercial reagents but a fully-effective neutralizer is still a challenge for researchers, thus promising candidates remain in the research phase.

## Introduction

Treatment with anticoagulants is associated with the risk of bleeding that increases with the dose, ageing, and concomitant administration of drugs affecting hemostasis, for example, antiplatelet agents [1–5]. Anticoagulant therapy is based on the administration of heparins, including unfractionated heparin (UFH) and low-molecular-weight heparins (LMWHs), and oral anticoagulants, such as vitamin K antagonists (VKAs) and direct oral anticoagulants (DOACs). VKAs may affect diagnostic tests and the reversal of their activity could be achieved by e.g., discontinuation of therapy or administration of vitamin K [6]. However, there are no reports the neutralization of warfarin in assays. The effect of UFH varies between patients, the adjustment of UFH dose and the monitoring of its effect are required [7]. The therapy with newer anticoagulants such as LMWHs and DOACs does not require routine monitoring. The assessment of LMWHs plasma concentration may be indicated in obese or pediatric patients, during pregnancy, or in those with impaired renal function [8, 9]. There are also many situations in which the measurement of DOACs activity is still necessary to make suitable decisions on patient management. The update of the International Council for Standardization in Haematology (ICSH) Recommendations from 2021 divides indications for monitoring DOACs into non-urgent and urgent situations [5]. DOAC therapy should be stopped for 2 to 3 days prior drawing of blood to minimize anticoagulant interference during testing. However, it is not always possible because of the risk of thrombosis [10]. Heparins and DOACs can affect the result of almost every coagulation test, thus precise monitoring of hemostasis in heparinized samples is not possible [10–15].

Accordingly, this narrative literature review examines the clinical and experimental literature and current guidelines regarding the removal or neutralization of DOACs and heparins in laboratory tests indicated in different clinical situations, for example during thrombophilia screening.

### Assays affected by anticoagulants and clinical necessity of their removal in diagnostic tests

Routine coagulation tests, such as activated partial thromboplastin time (aPTT), prothrombin time (PT), and thrombin time (TT) are clot-based activity assays used for the general assessment of coagulation function. The prolonged coagulation time may be associated with clotting factors deficiencies and/or the presence of their inhibitors. The influence of anticoagulants on routine assays is widely known. The aPTT and TT are sensitive to the presence of UFH, whereas PT is not affected by UFH and LMWHs. The LMWHs influence on aPTT depends on reagent sensitivity and plasma concentration of LMHWs. Among DOACs, dabigatran shows the strongest effect on aPTT and TT, while rivaroxaban on PT. The anti-factor Xa (anti-FXa) assay is very sensitive to the presence of direct FXa inhibitors and LMWHs [8]. Therefore, some of these tests are used to monitor anticoagulant therapy, such as aPTT for UFH, anti-FXa assay for LMWHs and direct FXa inhibitors [15–19]. However, when hemostasis disorders are diagnosed in patients, the presence of anticoagulant in blood samples may lead to inaccurate results. Viscoelastic tests, thromboelastography (TEG) and rotational thromboelastometry (ROTEM) provide an assessment of coagulation and fibrinolysis in whole blood. Compared to standard coagulation assays, they may detect cellular interactions to better reflect in vivo hemostasis [20]. High or pathologically low results of thrombin generation (TG) assay may inform about the risk of thrombosis or bleeding, respectively [21, 22] The presence of any anticoagulant inhibits TG [23].

Thrombophilia is hereditary or acquired condition characterized by an increased tendency to blood clotting which is associated with the occurrence of venous thromboembolism (VTE) [24]. Many guidelines do not recommend thrombophilia testing because of its limited clinical utility [25, 26]. However, recent National Institute for Health and Care Excellence (NICE) VTE guidelines suggest considering testing when it is planned to stop anticoagulation treatment in patients who have had unprovoked VTE and/or have a first-degree relative with an unprovoked VTE [27]. Screening for inherited thrombophilia is based on assays estimating deficiencies of the natural anticoagulant activity – antithrombin (AT), protein C and protein S [28, 29]. AT is an endogenous anticoagulant which inhibits several clotting factors. The deficiency of AT may be detected with AT assays, functional or immunological. Functional AT assays are based on the inhibition of factor IIa (FIIa) or FXa by AT in the presence of heparin. However, heparin therapy can lead to reduction (up to 30%) in AT levels [30]. Direct FIIa or FXa inhibitors, may falsely elevate the results of anti-FIIa and anti-FXa assays, respectively [8, 31]. Activated protein C resistance (APC-R) induced primarily by the factor V Leiden mutation increases the risk of thrombosis [28, 32, 33]. APC-R may be detected when a ratio between a baseline aPTT and aPTT after the addition of exogenous activated protein C is not prolonged/less than 2.0 [32–34]. Genetic determination is recommended after the positive results of APC-R which may be caused by all DOACs [35, 36]. Antiphospholipid syndrome (APS) is an acquired thrombophilia characterized by venous and/or arterial thromboses or pregnancy morbidities such as miscarriages and late intrauterine fetal demise [37, 38]. The laboratory criterium of APS is the presence of at least one of the antiphospholipid antibodies: lupus anticoagulant (LA), anticardiolipin (aCL) or anti-β_2_-glycoprotein I (aβ2GPI) [39, 40]. LA is detected by prolonged coagulation times. The aCL and aβ2GPI antibodies are identified by measuring immunologic reactivity to a phospholipid (cardiolipin) or a phospholipid-binding protein (β_2_-glycoprotein I) in immunoassays [41]. Because the presence of LA strongly correlates with clinical symptoms, the assessment of LA is useful for the diagnosis and management of APS patients [42]. According to the International Society on Thrombosis and Haemostasis (ISTH) guidelines, the detection of LA requires performing two tests based on different principles – the dilute Russell Viper Venom time (dRVVT) and an LA-sensitive aPTT (aPTT-LA) [42, 43]. The dRVVT relies on the ability of Russell’s venom to directly activate FX, while the aPTT-LA is based on the activation of the intrinsic pathway. The dRVVT appeared to be more sensitive to interference by DOACs than aPTT-based assays [44–46]. LA-positive patients should immediately start anticoagulation therapy, but the results of LA tests repeated after 12 weeks may be hampered by the anticoagulation treatment [26, 47, 48]. Seheult et al. in a large, retrospective study showed higher positivity rates of LA assays in patients treated with DOACs (> 50%) compared to patients treated with heparin (30–36%) [49]. Heparins may be administered in some cases, such as pregnant women with a history of obstetric APS or catastrophic APS [50, 51]. Both UFH and enoxaparin interfere with dRVVT and aPTT-LA assays [42, 51]. The 3-step procedure (screen-mix-confirm) allows for avoiding false-positive LA results in heparinized plasma. However, it is necessary to assess heparin levels before testing [42]. The neutralization of anticoagulants in patient’s blood samples may improve test results, and consequently allow correct diagnosis of thrombophilia.

The surgical interventions due to a bleeding risk often require the temporary discontinuation of DOACs, which may increase the risk of a thromboembolic event. Parenteral bridging relies on the use of short-acting anticoagulants such as heparin, although it is not usually recommended and reserved for patients with a high risk of thromboembolism [52–54]. A prophylactic dose of heparin may be helpful when reinitiating of DOACs needs to be delayed because of additional procedures or a postoperative patient’s oral medications intolerance [55, 56]. The ICSH guidelines recommend monitoring heparin bridging in DOAC-treated patients. However, the measuring of heparin activity by the assay e.g., aPTT, modified by both anticoagulants seems to be impossible but could be achieved after the removal of DOAC during assay performance.

Blood collection from a central venous catheter may yield plasma contamination of heparin, which is used to prevent catheter occlusion and infection [12, 57]. As Jeon et al. showed that even discarding a higher volume of blood than is recommended cannot avoid heparin contamination while blood collection from catheters [58]. We and others showed that the assessment of DOACs anticoagulant activity becomes impaired in heparinized plasma [13, 59]. The appropriate assessment of anticoagulant concentration or activity in plasma may be possible after the neutralization of coexisted heparin.

### Commercially available methods for DOACs removal in diagnostic tests

#### DOAC-Stop and DOAC-Remove

**Fig. 1:**
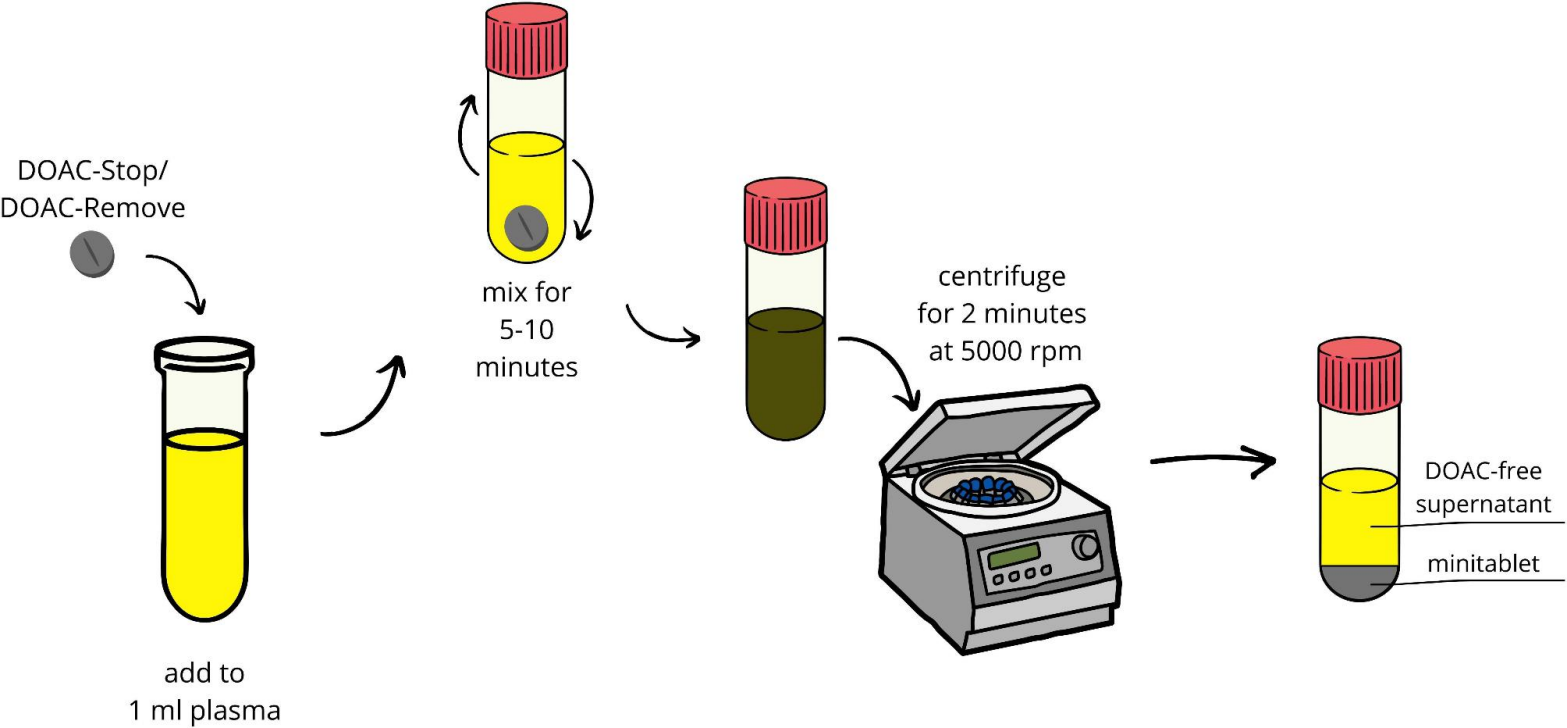
The procedure with using the DOAC-Stop/DOAC-Remove. DOAC, direct oral anticoagulant

The ability of activated charcoal to absorb contaminations or drugs has been used in binding agents – DOAC-Stop (Haematex Research) and DOAC-Remove (5-Diagnostics AG). According to the manufacturer, one tablet of DOAC-Stop or DOAC-Remove is sufficient to obtain plasma deprived of all DOACs (Fig. 1). Their absorbent properties have been reported in routine coagulation assays, such as aPTT, PT, TT, diluted thrombin time and fibrinogen Clauss [60–63]. However, Cox-Morton et al. noticed statistically significant removal of rivaroxaban and apixaban in PT in 17/20 and 13/20 samples, respectively. Besides, DOAC-Stop fully neutralized dabigatran in the factor VII (FVII), factor VIII (FVIII), and FX assays [60]. The efficacy of DOAC-Remove was confirmed in an anti-FXa assay [61, 62]. In the Jourdi et al. study, the total neutralization by DOAC-Remove reached 82% for apixaban and 98% for rivaroxaban in an anti-FXa assay [62].

Recent studies have proved the ability of DOAC-Stop and DOAC-Remove to eliminate protein C and S overestimation in the presence of DOACs [36, 46, 61]. Favresse et al. have demonstrated a significant decrease of APC-R after adding DOAC-Stop to samples with dabigatran and a smaller decrease in samples with edoxaban, while no decrease was observed in samples containing rivaroxaban and apixaban [36]. The lack of effect against rivaroxaban was probably due to using Pefakit APC-R factor V Leiden, which does not interfere which rivaroxaban [15, 64]. DOAC-Remove almost fully reduced the concentration of all tested DOACs allowing for the measurement of APC-R [35].

The latest 2020 updates of the ISTH guidelines for LA detection and interpretation recommended the use of DOACs neutralizers or adsorbents in DOAC-treated patients if suspending treatment is impossible [42]. However, it has been suggested that DOAC-Stop may prolong dRVVT and aPTT in patient samples without anticoagulants or with heparin [48]. Some studies reported incomplete DOACs removal by DOAC-Stop in aPTT-LA and dRVVT tests [65, 66]. Similar results were obtained in studies using DOAC-Remove [61, 62]. In the tandem mass spectrometry (HPLC-MS/MS), Slavik et al. observed that DOAC-Stop near-total eliminates DOACs from patient plasma [67]. The concentrations of dabigatran, rivaroxaban and apixaban achieved a maximum of 2.7, 10.97 and 13.03 ng/ml, respectively. These residual amounts of DOACs did not interfere with LA testing. The gap between these studies may come from interlaboratory variation or the used protocol of binding agents. For DOAC-Remove, HPLC-MS/MS revealed almost complete elimination of dabigatran and rivaroxaban; residual concentration below lower limit of quantification was reached in 7/8 and 8/10 samples, respectively. Removal effectiveness was lower in apixaban samples and reached 5/10 samples [62].

The ability of DOAC-Stop and DOAC-Remove to neutralize apixaban, dabigatran, edoxaban and rivaroxaban was also confirmed in TG assay measured using calibrated automated thrombography (CAT) or TEG [63, 68]. DOAC-Stop induced a slight procoagulant effect, probably due to a small inhibition of tissue factor pathway inhibitor (TFPI).

#### DOAC Filter

**Fig. 2:**
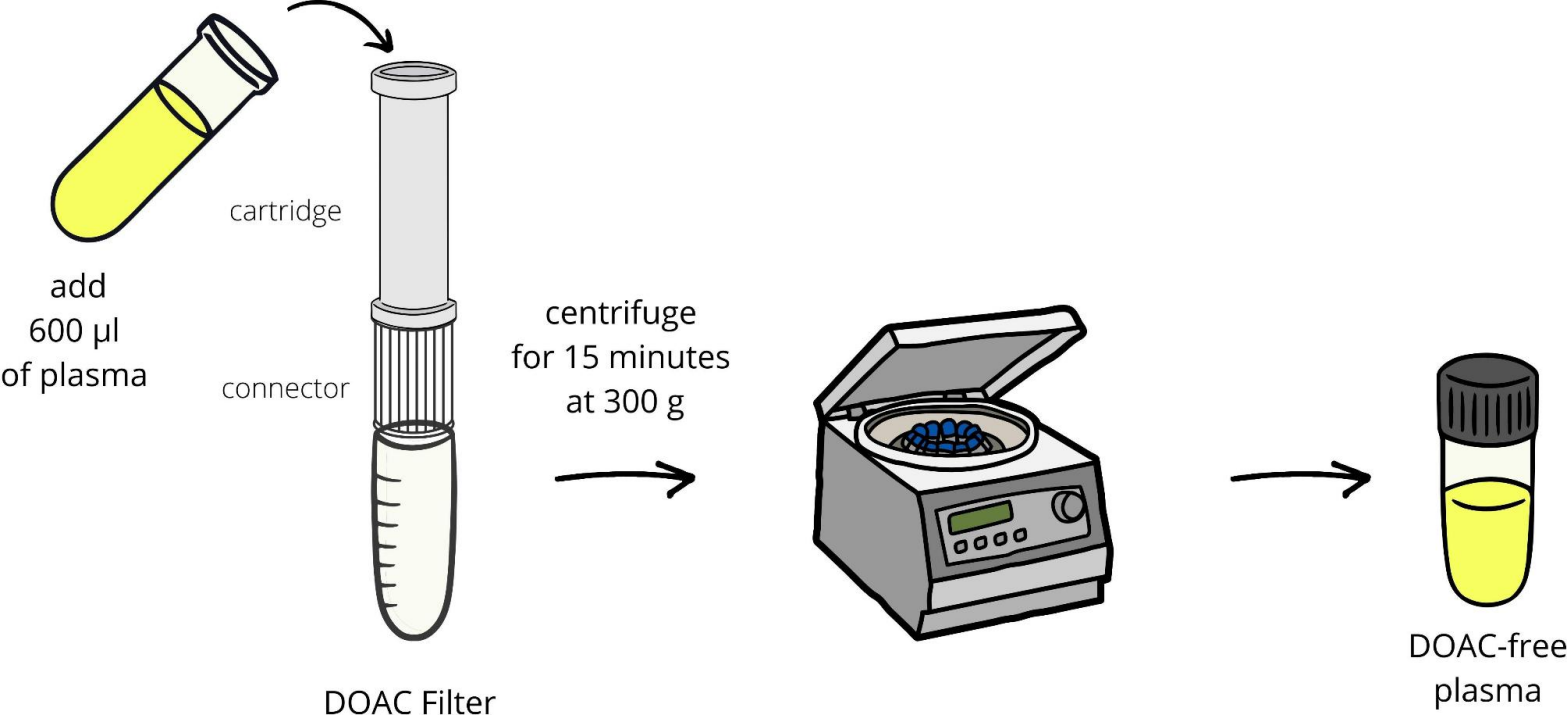
The procedure with using the DOAC Filter. DOAC, direct oral anticoagulant

The DOAC Filter (Diagnostica Stago) has been recently developed as a ready-to-use device with a filtration cartridge containing chemical hydrophobic-hydrophilic polymers to remove DOACs. It applies a solid phase extraction based on a noncovalent binding mechanism [69]. The sample of plasma is filtered and centrifuged (Fig. 2). Levels of DOAC are below the limit of detection after using DOAC Filter. The first studies showed sufficient removal efficiency in samples with rivaroxaban and dabigatran, while the absorption of apixaban-containing plasma was not complete [69–72]. A similar effect was observed in LA testing samples. In non-anticoagulated plasmas, some positive LA results changed into negative after DOAC Filter. During dRVVT and silica clotting time (SCT), interference of apixaban was removed in 50 and 60%, while for rivaroxaban in 84 and 83%, respectively. Accordingly to the ISTH guidance, using DOAC Filter should be limited to samples with DOAC [42, 72, 73]. DOAC Filter is a fairly new product, which requires further investigation to confirm its usefulness.

### Potential candidates for DOACs neutralization in diagnostic tests

#### Ciraparantag (PER977)

Ciraparantag (PER977; Perosphere Inc.) is a new small synthetic water-soluble molecule that is developed for the reversal of anticoagulants, including UFH, enoxaparin and DOACs (dabigatran, rivaroxaban, apixaban, and edoxaban), still under clinical trials [74]. Bonding between ciraparantag and anticoagulant relies on noncovalent hydrogen bonds and charge-charge interaction. No binding to FIIa and FXa, plasma proteins, or other drugs was found [75]. Lu et al. suggested a possible increase in human platelet activation by ciraparantag using P-selectin expression induced by 10 µM adenosine diphosphate [76]. In this study, ciraparantag did not bind DOACs in vitro, which may depend on the assay. Ansell et al. showed that the DOACs reversal effect of ciraparantag was dose-related and better against apixaban while the whole blood clotting time [77]. Because of high cationic charge and low molecular weight, ciraparantag could complex with anionic chemicals used in blood tubes, such as sodium citrate, ethylenediaminetetraacetic acid or activators used in coagulation assays (aPTT, PT, anti-FXa assay), as kaolin and celite. Thus, the investigation aimed to test its usefulness in diagnostic assays is required [75, 78].

#### Idarucizumab

Idarucizumab, a humanized monoclonal antibody fragment, was approved in 2015 as a reversal agent for dabigatran in patients with life-threatening or serious bleeding or requiring urgent invasive procedures [79]. The mechanism of action relies on the binding of free and thrombin-bound dabigatran and its active glucuronide metabolites by hydrophobic interactions, hydrogen bonds, and a salt bridge [79, 80]. Only few studies indicated the use of idarucizumab as in vitro neutralizer. Jacquemin et al. demonstrated that idarucizumab did not interfere with routine clotting times (aPTT, TT, and PT) and other coagulation assays (FVII, FVIII, FX assays, dRVVT, aPTT-LA) [81]. Recently, Mijovski et al. found that idarucizumab increased TG in samples without dabigatran [82]. Nonetheless, this antibody seems to be potentially useful in neutralizing dabigatran in assays. However, low stability, storage conditions and costs make routine use of idarucizumab in diagnostic tests questionable.

#### Andexanet alfa

Andexanet alfa, a recombinant modified inactive human coagulation FXa, is approved by the FDA in 2018 for the reversal of rivaroxaban or apixaban in life-threatening or uncontrolled bleeding [83, 84]. Andexanet alfa acts as a decoy molecule that competitively binds anti-FXa inhibitors, neutralizing their anticoagulant activity [84, 85]. A few studies proved that andexanet alfa did not have a procoagulant or anticoagulant activity in clotting assays, such as aPTT, PT and prothrombinase-induced clotting time [85–87]. Interestingly, Siddiqui et al. observed that in the whole blood clotting time only betrixaban from among all FXa-tested inhibitors was completely neutralized by andexanet alfa [87]. Although the inhibition of TG induced by FXa inhibitors was neutralized, adding andexanet alfa alone to plasma increased the TG. Favaloro et al. noticed that during LA-testing andexanet alfa was not able to fully correct aPTT and dRVVT in rivaroxaban-spiked samples [88]. The rivaroxaban-neutralizing effect by andexanet alfa was reagent-dependent and showed a higher spread of test data in comparison to DOAC-Stop during FVIII and factor IX (FIX) testing [89]. However, andexanet alfa does not impact coagulation proteins except transiently decreased activity of TFPI [84, 86]. In the presence of rivaroxaban, the andexanet-TFPI bond intensified TG [86]. Because of high-affinity binding to the drug-bound AT, andexanet alfa also effectively reverses the anticoagulant effects of ATIII-dependent FXa inhibitors, like heparins, which was confirmed by the results of coagulation tests [84–86, 90, 91]. Further investigation is needed due to limited clinical evidence [91]. The high price and mentioned impact on some assays hold andexanet alfa back for use in laboratory diagnostics.

### Commercially available agents for heparins removal in diagnostic tests

#### Heparinase

Heparinase is an enzyme obtained from the bacterium Flavobacterium heparinum. Its heparin-neutralizing abilities rely on the cleavage of alpha-glycosidic linkages at the ATIII binding site and prevent a heparin-AT-thrombin complexation [92, 93]. Lots of studies confirmed the reversal of the heparin effect by heparinase at aPTT, PT and TT tests [14, 94]. Heparinase restores the thrombin activity, decreases coagulation time and has a minimal effect on platelets [93, 95]. Current guidelines suggest the use of heparinase to quench the activity of both UFH and LMWH in LA detection [73]. There are commercially available TEG cuvettes with heparinase, which can be used in patients who received heparin [96]. Coppell et al. demonstrated effective neutralization of UFH, LMWH and danaparoid by heparinase [96]. HEP-TEM is a standardized, validated laboratory reagent containing heparinase. The addition of HEP-TEM to the TG assay can neutralize prophylactic and therapeutic doses of UFH and LMWH [97]. Strickland et al. have developed a new 3-step laboratory test to monitor UFH dosing using heparinase in patients taking apixaban. The difference between the first and second results of anti-FXa activity measurement indicates only the contribution of DOAC because of the removal of heparin by heparinase [59]. This assay may help in quantifying heparin in the presence of DOACs. Unfortunately, this approach requires longer analytical time than standard tests.

#### Polybrene

Polybrene (hexadimethrine bromide), a stable quaternary ammonium salt, has been known as a heparin-neutralizer agent since 1953 [98]. The mechanism of action is similar to that of protamine and depends on the interaction of cationic groups with anionic heparin chains. This synthetic polycation reverses the effects of heparin in vitro and in vivo. In first studies with polybrene demonstrated its superiority over protamine in the neutralizing of heparin with less severe side effects [99]. However, the clinical development of polybrene was stopped following reports of acute renal failure, proteinuria, hypotension and increased pulmonary artery pressure found in patients [92]. Kikura et al. demonstrated the polybrene ability to neutralize heparin in activated clotting time in comparison to another neutralizer available then [92]. Currently, polybrene is commonly used in vitro and can bind heparins in blood samples during routine tests such as PT and aPTT [100]. Cumming et al. proved that 100 µg/ml of polybrene was able to completely neutralize 10 IU/ml of heparin [101]. We also confirmed the usefulness of polybrene in the monitoring of dabigatran activity by TT test in the presence of heparin [13]. A commercially available reagent from Haematex - the Heparin Resistant Recalcyfying Solution (HRRS) – contains the calcium salt solution with polybrene, which may be used in aPTT, surface active clotting test and kaolin clotting test. Polybrene as a heparin neutralizer is also used in commercial kits for LA detection. Jacobsen et al. confirmed that adding polybrene to a sample containing heparin in a concentration of up to 1.3 IU/ml enables assessment of the lupus ratio regardless of heparin presence [102]. The guidelines recommend the use of polybrene or other neutralizers while LA testing [73]. Schäfer et al. conducted thromboelastometric tests using ROTEM delta analyzers for the detection and differentiation of DOACs and VKAs. Polybrene was included in EXTEM and FIBTEM tests for heparins removal [103]. During the TG study, polybrene (0.025 mg/ml) was able to bind UFH and enoxaparin up to 1.0 and 1.2 IU/ml, respectively, and completely restore TG in a concentration-dependent manner [11]. However, higher concentrations of polybrene may inhibit TG [97]. Furthermore, the prolongation of TG lag time and time to peak observed after the addition of polybrene may result from its inhibition of tissue factor-dependent FVII activation [11].

### Potential candidates for heparins removal in diagnostic tests

#### Protamine sulfate

The oldest heparin reversal agent is protamine sulfate (PS), approved in 1939. PS is an arginine-rich protein sourced from the salmon fish sperm or produced through recombinant biotechnology [104]. The mechanism relies on the interaction between positive charged PS and polyanionic heparin, which create stable complex in a 1:1 ratio and thus PS displace ATIII from heparin complex [105]. One milligram of PS neutralizes 100 IU of heparin. PS normalized TT and anti-FXa activity (to 0 IU/ml) which was achieved at 0.6:1 ratios of PS to UFH [106]. Increasing doses of PS prolong clotting time tests, such as activated clotting time, PT and aPTT because of interference with coagulation factors and platelet function [107, 108]. Because neutralization by PS depends on the molecular weight of heparin, the reversal effect of LMWH is only partial [109]. A lower concentration of PS restored TG prolonged by UFH or LMWH up to 0.4 IU/ml [97]. Zmuda et al. found that PS incompletely reversed the prolongation of reaction time induced by heparin in the TEG method [110]. Although PS is still a neutralizer of heparin effects in clinical practice, its use in routine diagnostic tests seems to be inappropriate due to the activation of coagulation [111, 112].

#### Heparin-binding copolymer

A synthetic macromolecule named heparin-binding copolymer (HBC), a diblock polymer containing a neutral poly(ethylene glycol) block and a cationic poly(3-(methacryloylamino) propyl trimethylammonium chloride) block was developed previously by us for heparin and heparin mimetics complexation [113–115]. The ability of HBC to bind UFH was presented by colorimetric and optical methods. In rats, HBC neutralized the anticoagulant activity of UFH during aPTT testing with a ratio of 0.65 mg of HBC for 100 IU of UFH [113]. Full neutralization of enoxaparin, nadroparin, dalteparin, tinzaparin, and fondaparinux required different concentrations of HBC during the measurement of the anti-FXa activity [114]. Recently, we showed the effectiveness of HBC in heparin neutralization in vitro during DOACs measurement. Prolonged TT by both UFH and enoxaparin was restored in samples with dabigatran. We also proved the ability of HBC for heparin neutralization while measuring rivaroxaban activity by anti-FXa activity assay, although the approach still requires validation [13, 114].

#### Other cationic polymers

We previously found that modified dextran and chitosan can neutralize UFH and normalize aPTT and bleeding time in rats and mice models of thrombosis [116–118]. Among different polymers, the most active and safe reversal agent was Dex40-GTMAC3 [119, 120]. However, in vitro study showed that Dex40-GTMAC3 prolonged the aPTT above the concentration of 50 mg/ml [119].

There are also cationic polymers such as universal heparin reversal agent, dynamic covalent polymers, and others described by Bromfield et al., which could be useful as neutralizers in diagnostic tests, although further research is needed to check if their efficacy profile could be better than protamine [121–123].

## Perspectives

Our review describes the methods of anticoagulant neutralization in diagnostic tests. Other options are methods that are not sensitive to anticoagulant activity. For example, even the concomitant presence of heparin in blood samples allows for the determination of DOACs activity using liquid chromatography-mass spectrometry. However, it has limited usage in routine diagnostics because of its low availability, time-consuming process and expensive equipment requirement [124–127]. The new rapid test – DOAC Dipstick – provides DOACs detection in the urine. Heparins cannot interact with DOAC Dipstick’s test pads because of a lack of antithrombin in urine [128–130]. This method for DOACs measurement could be helpful if urgent administration of antidotes is needed. For heparin, no gold-standard methods were established. The implementation of assay-neutral methods in routine diagnostics could greatly improve the performance of not only coagulation assays but also many other diagnostic tests.

## Limitations

Differences in used protocol between laboratories may affect the results of described methods. The current approach focuses on neutralizing heparins or DOACs in laboratory assays which is not a problem in the case of VKAs. The monitoring of acute clinical settings requires quick and accurate assays. The guidelines suggest the use of DOAC neutralizers in standard practice. Commercially available methods, such as DOAC-Stop, DOAC-Remove, heparinase and polybrene, seem to be useful in standard laboratory tests. However, it will make the diagnosis of some disorders such as APS even more complex in urgent cases. Additionally, pretreatment with neutralizers can be advised only in anticoagulant-treated patients. More specific assays can accurately quantify drug levels, making them useful in important clinical situations. However, they are not available in all laboratories and require high level of expertise. Furthermore, if a neutralizer was added, some disturbances were described in specific assays like thrombin generation assays. Experimental methods, like idarucizumab, andexanet alfa, protamine sulfate and HBC have few studies confirming their usefulness in diagnostic assays.

## Conclusions

The interfering with diagnostic tests by anticoagulants is a well-known issue and depends on the type of drug and its concentration, type of assay, reagents and analyzer used [72]. In this review we summarized different methods for removal of DOACs and heparins in diagnostic assays, both commercially available (Table 1), and in the development (Table 2). DOAC-Stop, DOAC-Remove and DOAC Filter were developed to neutralize DOACs. However, the incomplete reversal effect was observed, especially during LA testing. Idarucizumab and andexanet alfa, the antidotes for DOACs, are administered to patients with life-threatening bleeding, although their effectiveness as neutralizers in diagnostic tests has not been confirmed. Synthetic compounds, like polymers, or based on activated charcoal, seem to be the most promising in the neutralization of DOACs. Heparin may change the test results which could lead to incorrect patient diagnosis and therapy [131, 132]. Despite lengthy preparation of a sample or interference with some tests, heparinase and polybrene are present for removal of heparin in commercially available reagents.

**Table 1:** Commercially available methods for removal of anticoagulants in diagnostic tests

DOACs				
Method	Experimental procedure	Tests	Removal of anticoagulants	
			Complete	Incomplete
DOAC-Stop	1 tablet for 1 ml of normal plasma spiked with DOAC or plasma samples from DOAC-treated patients*	aPTT	dabigatran[60, 63], rivaroxaban [63], apixaban [63], edoxaban [63]	rivaroxaban [65]*, apixaban [65]*
		PT	dabigatran[60, 63], rivaroxaban [63], apixaban [63], edoxaban [63]	rivaroxaban [65]*, apixaban [65]*
		TT		dabigatran [60]
		fibrinogen	dabigatran [60]	
		FVIII assay	dabigatran [60], rivaroxaban [89]	
		FVII assay	dabigatran [60]	
		FX assay	dabigatran [60], rivaroxaban [89]	
		dRVVT	dabigatran [36]*, [60], rivaroxaban [36]*, apixaban [36]*, edoxaban [36]*	rivaroxaban [65]*, apixaban [65]*
		aPTT-LA	dabigatran [36]*, [60], [66]*, rivaroxaban [36]*, [66]*, apixaban [36]*, edoxaban [36]*	apixaban [66]*
		APC-R	dabigatran [36]*, [60], apixaban [36]*, edoxaban [36]*	rivaroxaban [36]*
		Protein C		dabigatran [36]*, rivaroxaban [36]*, apixaban [36]*, edoxaban [36]*
		Protein S		dabigatran [36]*, rivaroxaban [36]*, apixaban [36]*, edoxaban [36]*
		Antithrombin activity		dabigatran [36]*, rivaroxaban [36]*, apixaban [36]*, edoxaban [36]*
		CAT	rivaroxaban [63], apixaban [63], edoxaban [63]	dabigatran [63]
		TEG		dabigatran [63], rivaroxaban [63], apixaban [63], edoxaban [63]
		HPLC-MS/MS		dabigatran [67]*, rivaroxaban [67]*, apixaban [67]*
DOAC-Remove	1 tablet for 1 ml of normal plasma spiked with DOAC or plasma samples from DOAC-treated patients*	dTT	dabigatran [61]*, [62]*	
		Anti-FXa assay	rivaroxaban [61]*, apixaban [61]*, edoxaban [61]*	rivaroxaban [62]*, apixaban [62]*
		dRVVT		dabigatran [62]*, rivaroxaban [62]*, apixaban [61]*, [62]*, edoxaban [61]*
		APC-R		dabigatran [35]*, rivaroxaban [35]*, apixaban [35]*
		HPLC-MS/MS		dabigatran [62]*, rivaroxaban [62]*, apixaban [62]*
DOAC Filter	600 µl of normal plasma spiked with DOAC or plasma samples from DOAC-treated patients* loaded in the cartridge	aPTT	dabigatran [71], rivaroxaban [71]	apixaban [71]
		PT	dabigatran [71], rivaroxaban [71]	apixaban [71]
		dTT	dabigatran [72]	
		ECA	dabigatran [69]	
		Anti-FXa assay	rivaroxaban[69, 72], apixaban [69], edoxaban[69, 72]	rivaroxaban [70]*, apixaban [70]*, [72]
		SCT		rivaroxaban [70]*, apixaban [70]*
		dRVVT		rivaroxaban [70]*, apixaban [70]*
		HPLC-MS/MS	rivaroxaban [70]*, apixaban [70]*	
Heparins				
Heparinase	Added in the concentration of 2 IU[16, 99]/4 IU [99] to normal plasma spiked with heparin or plasma samples from heparin-treated patients*	aPTT	UFH [14]*, [59]*, LMWH [14]*	UFH [94]*
		PT		UFH [14]*, [94]*, LMWH [14]*
		TT	LMWH [14]*	UFH [14]*
		Anti-FXa assay	UFH [59]*	
		TGA		UFH [11], LMWH [11]
	Heparinized plasma samples added to heparinase I-coated plastic cuvettes	TEG	UFH [96], LMWH [96]	
Polybrene	Added in concentration of 0-100 µg/ml [13, 15, 97, 108–110] to normal plasma spiked with heparin or plasma samples from heparin-treated patients*	aPTT	UFH [101, 102]	UFH [100]
		PT		UFH [100]
		TT	UFH [102]	UFH [13], LMWH [13]
		ACT	UFH [92]*	
		Anti-FXa assay		UFH [13], LMWH [13]
		dRVVT	UFH [102]	
		TGA	UFH [11], LMWH [11]	

If anticoagulant removal was 100% or as defined by the authors, it was marked “complete”; if anticoagulant removal did not reach 100% or as defined by the authors, it was marked “incomplete”. References are given in parentheses. References with asterisk describe the effect of samples from DOAC or heparin treated patients. ACT, activated clotting time; APC-R, activated protein C resistance; aPTT, activated partial thromboplastin time; aPTT-LA, lupus anticoagulant sensitive activated partial thromboplastin time; CAT, calibrated automated thrombography; DOACs, direct oral anticoagulants; dRVVT, dilute Russell Viper Venom time; dTT, diluted thrombin time; ECA, ecarin chromogenic assay; HPLC-MS/MS, high-performance liquid chromatography-coupled tandem mass spectrometry; LWMH, low-molecular-weight heparin; PT, prothrombin time; SCT, silica clotting time; TEG, thromboelastography; TGA, thrombin generation assay; TT, thrombin time; UFH, unfractionated heparin

**Table 2:** Experimental methods for removal of anticoagulants in diagnostic tests

Method	Experimental procedure	Tests	Effectively removed anticoagulants	
			Complete	Incomplete
Idarucizumab	Added in concentration of 125 µg/ml[86, 87] to normal plasma spiked with DOAC	aPTT	dabigatran[60, 81]	
		PT	dabigatran[60, 81]	
		TT	dabigatran [81]	dabigatran [60]
		dRVVT, aPTT-LA	dabigatran[60, 81]	
		APC-R	dabigatran[60, 81]	
		FVII, FVIII, FIX and FX assays	dabigatran[60, 81]	
	Added in concentration of 125 µg/ml [87] to plasma samples from DOAC-treated patients*	TGA		dabigatran [82]*
Andexanet alfa	Added in concentration of 100 µg/ml [92], 200 µg/ml[93, 94] to plasma spiked with DOAC	ACT	betrixaban [87]	edoxaban [87]
		aPTT	rivaroxaban [87], edoxaban [87], betrixaban [87]	apixaban [87]
		PT	rivaroxaban [87], apixaban [87], edoxaban [87], betrixaban [87]	
		PiCT		rivaroxaban [87], apixaban [87], edoxaban [87], betrixaban [87]
		Anti-FXa assay		rivaroxaban [87], apixaban [87], edoxaban [87], betrixaban [87]
		dRVVT, aPTT-LA		rivaroxaban [88]
		FVIII, FIX assays		rivaroxaban [89]
		CAT	rivaroxaban [87], apixaban [87], edoxaban [87], betrixaban [87]	
Protamine sulfate	Added in concentration of 0-200 µg/ml [109, 117, 118] to normal plasma spiked with heparin or plasma samples from heparin-treated patients*	aPTT		UFH [101, 106]
		PT		UFH [106]
		TT	UFH [106]	
		Anti-FXa assay	UFH [106]	UFH [109], LMWH [109]
		Anti-FIIa assay		UFH [109], LMWH [109]
		FVIII, FIX assays		UFH [106]
		TEG		UFH [110]*, LMWH [110]*
Heparin-binding copolymer	Added in concentration of 10–50 µg/ml [15] to normal plasma spiked with heparin	aPTT	UFH [113]	
		TT		UFH [13], LMWH [13]
		Anti-FXa assay	LMWH [114]	UFH [13], LMWH [16, 113]

If anticoagulant removal was 100% or as defined by the authors, it was marked “complete”; if anticoagulant removal did not reach 100% or as defined by the authors, it was marked “incomplete”. References are given in parentheses. References with asterisk describe the effect of samples from heparin treated patients. ACT, activated clotting time; APC-R, activated protein C resistance; aPTT, activated partial thromboplastin time; aPTT-LA, lupus anticoagulant sensitive activated partial thromboplastin time; CAT, calibrated automated thrombography; DOAC, direct oral anticoagulant; dRVVT, dilute Russell Viper Venom time; LWMH, low-molecular-weight heparin; PiCT, prothrombinase-induced clotting time; PT, prothrombin time; TEG, thromboelastography; TGA, thrombin generation assay; TT, thrombin time; UFH, unfractionated heparin

**Fig. 3:**
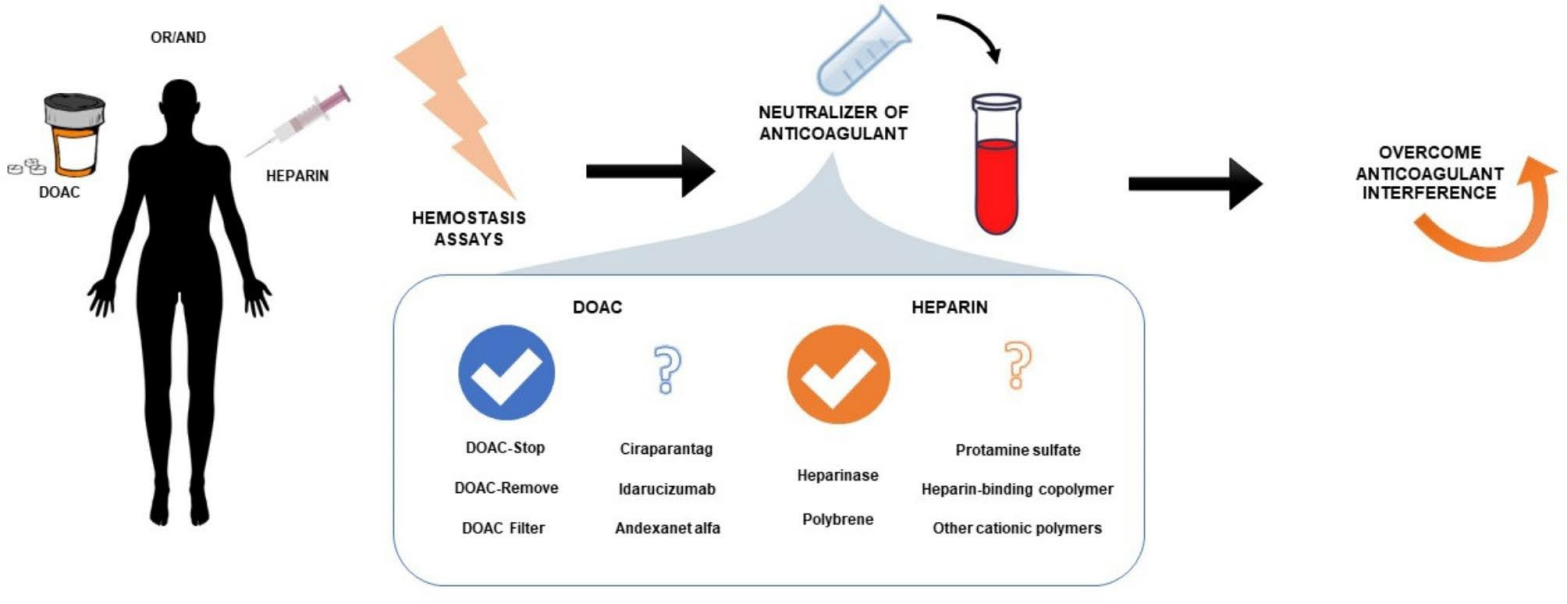
Graphical conclusions from the study. DOAC, direct oral anticoagulant

Whenever hemostasis is monitored, the results can be affected by the presence of anticoagulants in the blood sample. Particularly, the situations when the contamination of sample is unknown or not expected, for example in unconscious patient, are the most vulnerable to misinterpretations. Thus, the elimination of anticoagulants from sample could improve the reliability of assay, and have potentially broader application (Fig. 3). The incomplete reversal action, interference with reagents/assays or neutralization of only one type of anticoagulant are drawbacks of currently available methods. A whole lot of potential candidates have not been studied yet.

## Data Availability

Not applicable.
